# ‘The Emperor's new clothes?’ Healthcare professionals’ perceptions of the nursing associate role in two UK National Health Service hospitals: A qualitative interview study

**DOI:** 10.1016/j.ijnsa.2024.100211

**Published:** 2024-06-06

**Authors:** Carolyn Spring, Enrique Castro-Sánchez, Mary Wells

**Affiliations:** a Centre for Nurse, Midwifery and Allied Health Professionals Research, UCLH, UK; b Imperial College Healthcare NHS Trust, UK; c Brunel University London, UK; d Imperial College London, UK

**Keywords:** Health care organisations, Health care policy, New nursing roles, Nursing workforce, Scope of practice, Staffing deficit, Staff retention

## Abstract

**Background:**

The introduction of nursing associates in England in 2017 as a professional ‘bridging’ role aimed to mitigate chronic staffing shortages, enable career progression of healthcare assistants and release registered nurses to provide more complex care. Limited evidence exists about the alignment between the identity and purpose of nursing associate roles described by the UK independent regulator, the Nursing & Midwifery Council, and the expectations, obligations, and team dynamics encountered in practice.

**Purpose:**

Investigate the perceptions of nursing associate roles through the views and experiences of role holders, registered nurses, and healthcare assistants.

**Setting:**

Two British National Health Service (NHS) Hospital Trusts in London, England (UK).

**Methods:**

For this registered service evaluation, data were collected via in-person, semi-structured interviews. Verbatim transcripts were coded inductively. An adapted framework analysis method, suitable for use with Excel, was applied to support the identification of cross cutting themes. We used the Standards for Reporting Qualitative Research checklist for reporting this study.

**Results:**

Eleven registered nurses, five nursing associates, and five healthcare assistants participated. Their experiences seldom reflected the policy vision of the nursing associate role in practice. Several participants likened the nursing associate role to the fable of the ‘Emperor's New Clothes’ in which expectations and reality diverge. With this over-arching theme, four sub-themes were identified: (1) preparedness of organisational infrastructure to support this role; (2) credibility of the role in practice; (3) perceived organisational “blindness” to the ambiguities of the role and (4) increasing task orientation and segmentation in care delivery.

**Conclusion:**

There is a discrepancy between the identity of the nursing associate role as imagined in the policy agenda and its reality in practice. There is a need for more protected and well-defined training, clear role boundaries, and accessible career progression pathways for nursing associates. Moreover, honest dialogue at an organisational and policy level must continue, so that the challenges and opportunities of the nursing associate role are properly realised.

**Tweetable abstract:**

Emperor's new clothes! Experiences and views of new nursing associate roles in NHS (UK) acute hospitals @CarolynSpring3.

**What is already known?**

• Poorly defined role parameters, unmet training needs, excessive role expectations, and unreasonable workloads impede the introduction of new roles in practice.
• There is limited evidence to guide the implementation, evaluation, and optimal utilisation of the new nursing associate role.
• The views and experiences of nursing associates are largely unexplored.

**What this paper adds**

• There is a discrepancy between the identity of the nursing associate role as envisaged in the policy agenda, and its reality in practice.
• Staffing shortfalls and burgeoning workloads led to a blurring of nursing workforce roles.
• Role holders were perceived to predominantly function in a nurse capacity to meet unmet needs.

## 1 Introduction

The global nursing deficit is a worldwide health emergency linked to insufficient investment in nurse education, inadequate long-term workforce planning, unsustainable reliance on international recruitment, and failure to co-ordinate policy and planning intra- and internationally (Buchan and Catton, 2023). Strategies to address this deficit include encouragement for nurses to return to practice, supporting recruitment and retention, appointing overseas nurses (Peters, 2023) and increasing use of second-tier nursing roles (Lucas et al., 2021).

The nursing structure in the United Kingdom (UK) prior to 2000 comprised two levels, the state registered nurse (SRN) and the state enrolled nurse (SEN). The latter was phased out in the 1990s, reducing workforce numbers and increasing the skills gap between registered nurses and healthcare support workers, who are unregistered, but undertake a standardised training to achieve a care certificate (Glasper, 2016). Systemic failings of care (Francis Report, 2013) led to a review of such roles (Cavendish, 2013) and a re-examination of nursing education and training (HEE, 2016).

The second-tier nursing associate role introduced in 2017 by Health Education England aims to alleviate shortfalls from 43,000 unfilled nursing vacancies in the British National Health Service (NHS) in England and Wales. Designed to be both a standalone role and a new route to attain registered nurse status, it seeks to bridge the skills gap by progressing unregistered and unregulated healthcare assistants into graduate level nursing roles and enabling registered nurses to undertake more complex care (HEE, 2016).

Following pilot testing in 35 English sites, trained nursing associates gained registration with the UK nursing regulatory body (Nursing & Midwifery Council, NMC) in 2019. Apprentice nursing associates now complete a Nursing & Midwifery Council-approved pre-registration Foundation degree, comprising a salaried two-year vocational education programme integrated with clinical practice (HEE, 2016). Following this programme, nursing associates can further achieve nursing status via completion of a shortened nursing course (Table 1 for current NHS nursing workforce structure). Qualified proficiency standards for registered nursing associates span six platforms, differing from those for registered nurses (RNs) in leadership responsibilities (Table 2).

**Table 1:** NHS workforce structure, England and Wales.

Healthcare/nursing assistants	
Band 2	junior healthcare assistant (HCA)
Nursing associates	
Band 3	senior healthcare assistant / nursing associate apprentice (ANA)
Band 4	registered nursing associate (RNA)
Nurses	
Band 5	registered nurse (RN)
Band 6	experienced nurse
Band 7	specialist nurse, nurse with leadership responsibilities, advanced practitioner, ward nurse
Band 8	nurse with significant leadership or management responsibilities, matron

**Table 2:** Nursing and midwifery council (NMC) roles, registered nursing associates and registered nurses.

Registered Nursing Associate	Registered Nurse
6 Core responsibilities	7 Core responsibilities
1. Be an accountable professional	1. Be an accountable professional
2. Promoting health and preventing illness	2. Promoting health and preventing illness
3. Provide and monitor care	3. Provide and evaluate care
4. Working in teams	4. Leading and managing nursing care and working in teams
5. Improving safety and quality of care	5. Improving safety and quality of care
6. Contributing to integrated care	6. Coordinating care
	7. Assessing needs and planning care

By March 2024, more than 10,000 nursing associates were on the Nursing & Midwifery Council register in England (NMC, 2024). The intention was to train 5000 apprentice nursing associates in 2024/25, increasing to 7000 a year by 2028/29. It is anticipated that over 64,000 registered nursing associates will be employed in the NHS by 2036/37 (NHS England, 2023). The Nursing & Midwifery Council has recently approved an expansion of the role into Wales following a request from the Chief Nursing Officer for Wales (NMC, 2024). The Nursing & Midwifery Council *Standards of Proficiency for Nursing Associates* identify the general criteria and expectations for the role (NMC, 2018a,b; Table 2), but do not include guidelines for specialist areas, such as critical care, and there is significant organisational discretion to tailor the position to area-specific requirements. The independent healthcare regulator (the Care Quality Commission) is similarly not prescriptive about role deployment (CQC, 2019).

The barriers and facilitators to the implementation of new roles in health care are well documented. Poorly defined role parameters, unmet training needs, and lack of understanding of the new role are known to impact workplace integration (Robert et al., 2019). Excessive role expectations and unreasonable workloads also hinder employee transition (Kim and Shin, 2020). Embedding new roles in practice can be facilitated by clearly delineated roles and responsibilities between different levels of staff seniority and expertise (Henshall et al., 2018), and effective supervision and leadership (Hasle et al., 2018). Career development pathways and timely access to appropriate education and training can also support staff development and retention (Attenborough et al., 2020).

Currently limited evidence exists to guide the implementation, evaluation and optimal utilisation of the apprentice and registered nursing associate’ role. Initially, evaluation focused primarily on training and development programmes for apprentice nursing associates (Traverse, 2018, 2019) rather than how the role was performed and perceived in practice. Later studies identified challenges relating to role ambiguity, role clarity and establishing a career pathway as a registered nursing associate. (Kessler et al., 2020, 2022; King et al., 2020). Insufficient planning, poor communication, and a lack of organisational preparedness were also identified as barriers to registered nursing associate role implementation (Lucas et al., 2021) Yet little is known about stakeholders’ perceptions of registered nursing associates and the experiences of registered nursing associates or if they align with the Nursing & Midwifery Council's expectations for the role. Neither it is known how registered nursing associates feel about the knowledge and skills they acquire in practice and the level of support available to advance their career aspirations. An improved evidence base would allow for better understanding of the barriers and facilitators to role integration, so that these factors can be addressed in practice.

## 2 Methods

This study, conducted between May 2021 to September 2022, was registered as service evaluation number 543 with the NHS Trust Joint Research Office and Audit Office. Semi-structured one-to-one interviews were undertaken with three professional groups—healthcare assistants, registered nursing associates, and registered nurses—guided by a phenomenological approach (Neubauer et al., 2019) to support understanding of participants' experiences. In addition, an adapted framework analysis method (Swallow et al., 2003) was utilised to manage and organise the data

Participants were recruited from different hospital sites within two London NHS hospitals. The study was promoted to potential participants via hospital newsletters and emails cascaded through matrons, practice educators, and ward managers. A participant information sheet and consent form were provided to individuals expressing interest, with opportunities to request further information. Interviews were then arranged at a suitable time and date, and conducted in person in private spaces, or via Microsoft Teams. The study aimed to include 10 individuals from each professional group. The interview topic guide is shown in Table 3.

**Table 3:** Topic guide for semi-structured interviews.

Focus	Indicative topic for nursing associate interviews	Indicative topic for interviews with registered nurses and healthcare assistants
Introduction	Initial attraction/motivation to become	Familiarity with the nursing associate role Level of interaction with nursing associates in practice
Experiences	Most/least crucial elements of the role in training/practice Emotions experienced in conducting role	Type of interactions with nursing associates Attitudes and practices of nursing associates observed in care interactions with patients
Learning/work/balance	Balance between education and practice needs Ability to apply and use taught skills in practice area/s Experience of transitioning from trainee to qualified registered nursing associate status	Opportunities and scope for nursing associates to engage in learning observed in area Approach to learning by nursing associates Match between learning and practice requirements
Team / colleagues interaction	Attitudes and approach towards role from registered nurses and healthcare assistants Barriers and facilitators to collaborative working practice Designated responsibilities and tasks	Observed attitudes and approaches of nursing associates with healthcare assistants and registered nurses and the member of the wider clinical team
Professional identity	Self-perception/ perceived by others. Individual nature of your role Nature of your role within the care team	Level of understanding and recognition of the professional identity of the nursing associate within the clinical area
Organisational factors	Understanding of colleagues of your role Impact of leadership/ supervision on role Impact of teamwork/individualism in care delivery	The experience of leading/supervising/supporting nursing associates The impact of nursing associates on the organisation of care delivery and care outcomes Senior Trust leadership communication relating of the nursing associate role
Professional development/future goals/longer term plans	Development of skills and experiences Intention to become registered nurse? Specialise in specific clinical area? Work in a community setting	Types of interaction regarding the ambitions and career goals of nursing associates Views of nursing associates progressing to registered nurses/specialising Appropriateness of nursing associates in high acuity settings/community settings/specialising in specific areas?

The main researcher took reflexive notes to encourage self-awareness during the study and to consider subjectivity in relation to their emotional response and psychological interpretation. All interviews were transcribed verbatim and proof read several times to ensure accuracy. Participants' transcripts were anonymised with a unique identification number, and their responses pasted into Excel. An adapted framework analysis for use with Excel was selected (see Table 5), guided by Swallow et al. (2003).

Following re-reading for familiarity, preliminary notes were made, and phrases or sentences highlighted prior to assigning ‘codes’ to describe their content. Initially these data were coded inductively from the transcripts within each professional group. This process involved a combined approach (Skjott Linneberg and Korsgaard, 2019), applying ‘in vivo’ coding directly to the words of participants and ‘values’ coding to understand their perceptions and views of the registered nursing associate role. ‘Structural’ coding was employed to establish who performed specific actions (when and where), and ‘descriptive’ codes were used to determine the tasks undertaken. (Table 4).

**Table 4:** Example of the coding process.

Interview conversation extract	Initial coding	Values/valuing/value laden	Structural	Descriptive
“I am *not sur*e (1) [name redacted] [the registered nursing associate] *is any different* from our nurses (2). She seems to do the *same* (2) *tasks (3) the meds, the IVs,* [..]*, the same workload (4). [..]* [Redacted] [NHS Trust] *probably have their reasons (5) why, for pushing this [role]. They (NAs) are seen like the band* 5 s *[registered nurses]. [..] I don't really buy it (6) [..] Its quite convenient (7).[…] What's that old story?* Um, the king. No. *It's the Emperor. The Emperor's new Clothes (8). Yeah.* That's where it is at.	1. uncertainty 2. similarity 3. task-based 4. work volume 5. hidden/not visible 6. doubt/disbelief 7. expediency 8. Metaphor – pretence	I don't really buy it. convenient Old story Pushing Emperor's new clothes	same tasks on ward same workload in the clinical area	Meds IVs [intravenous therapy]

Codes were identified, added and revised and finally condensed to summarise key points. Then, responses from apprentice and registered nursing associates, healthcare assistants, and registered nurses were contrasted between and within cases. Finally, using an iterative approach, themes were determined through recurrence and alignment of relevant codes. Preliminary themes were discussed with all authors within the study team, and revisions applied to the wording of themes (Table 5).

**Table 5:** Adapted framework analysis method.

1	Familiarisation—immersion in the data	Listening to the recorded interviews and reading. Highlighting the emergent data listing key words, experiences, recurrences and divergences in views and experiences.
2	Developing a framework for analysis	Inductively coding the data into recognisable elements Revision of coding of data based on re-readings Developing themes arising from recurring views and experiences Labelling data into categories within and across professional groups
3	Indexing and identifying subthemes:	Codes were grouped into themes and distinct subthemes within each of the themes. Following completion of indexing, the indexed documents were reread and developed into thematic charts.
4	Charting the data into a framework	Use of Excel's reviewing tool bars to comment and colour code cells to chart the data and compare the experiences of the three professional groups and identify wider concurrence and divergence between and within professional groups to help achieve agreement of theme wording.
5	Mapping and interpretation	Checking and comparing themes and subthemes against the original transcripts, notes and audio recordings to see if any additional changes or merging was required. Agreeing final framework, themes and subthemes.

## 3 Results

Twenty-five participants expressed an interest in participation, four of whom subsequently declined. These included one registered nurse who dropped out based on lack of familiarity with the registered nursing associate role and two healthcare assistants and one registered nursing associate who chose not to take part due staffing issues in their work area. Twenty-one participants were interviewed, including 11 registered nurses, one apprentice nursing associate, four registered nursing associates and five healthcare assistants. Participants in all groups had different age ranges and ethnicities; however, the registered nurse group included a greater number of white nurses in senior bands (Table 6). Individuals working within both adult and children's areas were interviewed. Their clinical areas ranged from general surgery and medicine to critical care and emergency departments. The interviews averaged 49 minutes (range 34 to 81 minutes).

**Table 6:** Characteristics of study participants.

Gender (n)	
Men	4
Women	17
Ethnicity	
Participants from minority ethnic backgrounds	59%
Participants identifying as white	41%
Percentage of white nurses in senior bands 7-8	71%
Age range of participants (n)	
20-29 years old	4
30-39 years old	6
40-49 years old	5
50-59 years old	4
60-69 years old	1
Professional role and NHS banding of participants (n)	
Junior Healthcare Assistant Band 2	1
Senior Healthcare Assistant Band 3	4
Apprentice Nursing Associate Band 3*	1 (newly qualified - completing to Band 4 <5 weeks during the study inception)
Registered Nursing Associate Band 4	4 (5 months to 19 months registration experience).
Registered Nurse Band 5	2
Experienced Registered Nurse Band 6	2
Specialist Registered Nurse leaders (Ward Manager) Band 7	3
Registered Nurse senior leaders (Matron) Band 8	4

* Achieved nursing associate registration in duration of study.

Quotes are provided to illustrate each of the key sub-themes. The core theme of ‘the Emperor's New Clothes’ was derived from registered nurses (Bands 5, 6,7 & 8) and healthcare assistants (Bands 2 & 3) using this phrase in relation to the nursing associate role, referring to the 1837 Danish fable by Hans Christian Andersen (Box 1).

**Box 1. ‘The Emperor's New Clothes’**

The Emperor is misled by swindlers posing as tailors, who promise that the custom-made ‘clothes’ adorning him can only been seen by those fit for their posts. Shivering in his nakedness he publicly parades his ‘finery’, and his courtiers and the crowds play along, not wishing to appear stupid. Finally, a small child acknowledges the deception by shouting “He is naked!”.

The moral of the story is that pride, and fear of opposing popularist opinion must be succeeded by the truth and we should not accept that something is good because an “expert” says it is.

In the context of this study, the phrase signified that, in relation to registered nursing associates, the organisation, like the Emperor, may ‘see what they wish to see’ –a tailor-made effective bridge between healthcare assistant and registered nurse roles–, rather than openly acknowledging its limitations in scope, implementation, and effectiveness to meet clinical need. The registered nursing associates’ role expectations were not readily borne out in practice, so like the Emperor, their perception of how they appeared - what their uniforms should confer - often differed from the views of other professional stakeholders. Additionally, the four subthemes were: 1) preparedness of organisational infrastructure to support registered nursing associate role; 2) credibility of the role in practice; 3) perceived organisational “blindness” to the limitations and ambiguities of the role and, 4) increasing task orientation and segmentation in care delivery (Table 7).

**Table 7:** Key themes and sub-themes.

‘The Emperor's New Clothes’
1. Preparedness of organisational structure to support registered nursing associate role 1.1 Variable support for training and development in clinical practice 1.2. Sink or swim’—registered nursing associates determine their own passage 1.3. Presumption of supervision and support structure not fully evidenced 1.4. Nursing associates' experiences tempered by professionals’ expectations in practice
2. Credibility of the nursing associate's role in practice 2.1. Steppingstone for progression not a distinct professional identity 2.2. Blurring of roles in practice
3. Perceived organisational “blindness” to the ambiguities of the registered nursing associate role 3.1. Inadequate identification and acknowledgement of registered nursing associate roles 3.2. Registered nursing associates adapted to meet expectations of performing a hybrid nurse role
4. Task orientation—trend towards `taskification’ within care

### 3.1 ‘The Emperor's new clothes’

The overarching theme refers to participants’ feelings of having believed in, or even having been ‘duped’ about the defined professional identity of the registered nursing associate role, when in practice it seems to be more intangible. Indeed, tensions about the similarities in registered nursing associate and registered nurse roles, together with scarce human resources to implement the registered nursing associate role effectively, impacted the confidence about the credibility of registered nursing associate roles. The institutional propensity to “see what they wished to see” instead of these tensions was challenged.

“*I wonder that there is a wilful blindness here […] a whole mass of uncertainty. [..] I think the role has evolved to fill a sort of vacuum and things are thawed out as time progresses”. [..] They'll say, [..] “oh it is agile”, but [..]. Whose it agile for? Agile for the budget, that's who, not for all us lot. No. So, […] stick your [..] head in the sand, […] fingers crossed, eyes shut […] hope for the best.”* Registered Nurse 13 Band 8.

*I love my role. I do go above, yes, and beyond. [redacted Ward Managers name] knows that. Why would they say, “oh yes, she's a nurse”? Choose to see that*? *I don't think so”* Registered Nursing Associate 2, Band 4. (11 months qualified).

The registered nursing associate role was described using words such as ‘charade’, ‘illusion’, ‘fantasy’ and ‘fiction’ and hence likened by several participants to the Danish folktale.

“*There is a sense of what you could call the Emperor's New Clothes about nursing, you know, that if nursing associates are good enough to do all this work. [..] What's the difference? What is it that nurses have? What have the nurses got that makes them better paid or higher status or, you know, a bit sort of superior? It became slightly hard to say what it was, apart from their uniform*” Registered Nurse 16 Band 7

The tasks, duties and responsibilities of registered nursing associates were not well understood. Only one registered nursing associate reported receiving a job description. Registered nursing associate role holders did not feel their role was distinct from other nursing roles; rather, to fit the specific ward needs, they sometimes functioned largely as a healthcare assistant. Registered nursing associates undertook monitoring patients performed personal care and provided mealtime assistance, yet more commonly, they undertook tasks within the scope of a registered nurse, often without direct supervision. A senior nurse voiced that the current requirements to enter nurse education programmes played a part in sustaining this illusion:

“*This role is a pretence grade. You don't need people with degrees. They have made a rod for their own back, and they have got round it by getting in another position—it [the registered nursing associates role] is a nurse role*” Registered Nurse 13 Band 8

1. Preparedness of the organisational infrastructure to support the registered nursing associate role

Four sub-themes relating to organisation preparedness were identified from the interviews: (1.1) Variable support for training and development in clinical practice (1.2) ‘Sink or swim’—registered nursing associates determine their own passage. (1.3) Presumption of supervision and support structure not fully evidenced and (1.4) registered nursing associates's early expectations tempered by experience in practice.

1.1 Variable support for training and development in clinical practice.

Although widely welcomed by participants as a debt-free door to Nursing & Midwifery Council registration, apprenticeship training milestones relied on limited staff educators:

*“They just don't have the time to teach or supervise me. [..]. So, I would do the same tasks as a healthcare assistant. Again, and again. I did get some balance [..] from my own initiative. Yeah, learning had to come from me*”. Registered Nursing Associate 2 Band 4 (11 months registered)

Not all registered nurse participants valued this self-advocacy and proactiveness in learning; some admired apprentice nursing associates' resolve and initiative, while others viewed it as a result of a lack of organisational leadership:

*"It is such a top-down lack of knowledge, and it feels like a bottom-up drive [..] I wonder if that is what they are aiming for. Is it getting the nursing associates to push us to fight their way, emerge into their own sense of identity? But I think it is a top-down approach that needs to be taken. That is what is missing for me. Nobody is even willing to look at the job descriptions*”. Registered Nurse11 Band 8

This uncertainty, on the other hand, fostered flexibility to access learning opportunities beyond those mandatory for registration, aligned to the requirements of the specific clinical area:

“*I've had experiences on my placement, whereas a lot of people haven't had that experience. [..] Things like inserting catheters, which isn't in the proficiencies. It's another one of those lines of learning and tasks, that's blurred. But I have accomplished this*”. Apprentice Nursing Associate 3 Band 3 (in process of registration).

There were instances when it was apparent that the education and training provided did not match ward requirements. A registered nursing associate in a paediatric setting commented: "*I was the only [apprentice] nursing associate in a children's setting [..] even though a nursing associate is duly trained, we look after people of all ages, the actual foundation degree was 95 % adult based".*Registered Nursing Associate 5 Band 4 (5 months registered).

Efforts to strategically embed a supportive pathway for the registered nursing associates were sometimes frustrated through lack of leadership, interest, or competing directives.

“*I started the process, putting a business plan forward [..] to say where the nursing associate could be included and how many we could have on the wards to make it viable and truly effective. And what happened? The new [Leader in my area] came in and would not even read my strategy. And [..] just said “no that idea will not work”. And that was the last I heard of it*” Registered Nurse 11 Band 8

1.2 ‘Sink or swim’—registered nursing associates determine their own passage.

It was often stated by registered nurses and healthcare assistants that registered nursing associates appeared to be left to ‘sink or swim’ or ‘left to their own devices’. Both registered nurses and healthcare assistants identified that several were ‘buoyed up’ or ‘coped’ due to knowledge, skills and experience gained in previous healthcare roles:

“*I felt like they were just thrown in the deep sea, and okay, just learn how to swim. There wasn't clear guidance [..] or information from the beginning. [..] It was "so here they are, use them*”. Registered Nurse 17 Band 6

“*It's been a tough call [..] she knows the ropes, she started together in [..] in 2015, no, 2016. She's got these ambitions, yeah, and her experience, here, and in ENT [Ear, Nose and Throat], she's keeping up, She'll survive, I know it. She won't sink. We go back, you see”* Healthcare Assistant *7, Band 3.*

Prior experience was sometimes incorrectly assumed of the registered nursing associates; a registered nursing associate, who gained registration early on in the study, advised that it was ‘all uphill’ as she had to constantly reiterate her unfamiliarity with tasks requested to gain the appropriate guidance. To ‘soldier on’ was frequently used by participants to describe how registered nursing associates fulfilled their roles. Senior nurses noted how the workplace experiences of registered nursing associates were particularly challenging and attested to their resilience:

“*When they are thrown into an accident and emergency environment or an intensive care unit environment that became very difficult. They coped, but you could noticeably feel the stress pooling out of them. [..] But they soldiered on*" Registered Nurse 18 Band 8

1.3 Presumption of supervision support structure not fully evidenced.

For participating healthcare assistants and registered nursing associates, registered nurse support and supervision of registered nursing associates was variable. Some reported excellent supervision, whilst for others, it was given intermittently or reluctantly, or even largely absent, as noted by two registered nursing associates:

“*There seems to be a lot of discretion around people who've worked here longer or have some healthcare assistant experience and there's less supervision for us, but also generally, the supervision here is in fact quite poor”.* Registered Nursing Associate 4 Band 4 (9 months registered).

The demands of the ward, registered nurse inexperience and low confidence could impact the willingness and capacity of registered nurses to supervise their practice:

“*Well, the nurses will sometimes [..] say, well, I have my own set of patients. Why do I have to look after her? I don't need to be supervising other staff members. Why does [the registered nursing associate] get so much leeway? What after all the other things I've done? Or they are in that position of wait, I'm not experienced here, I need support so why the hell am I supporting someone else?* Registered nursing associate 2 Band 4.(11 months registered).

Confusion also prevailed on whether and when registered nursing associates should be expected to work independently. This uncertainty appeared to be linked to a lack of clarity stemming from limited direction towards defining role responsibilities:

*"I don't think there was sort of ownership on who defines the [registered nursing associate] role during practice. [..] It could get very blurred. It was not clear whose job it was to say, oh no, they shouldn't be doing that. Or yes, they should be doing that" Registered Nurse 15 Band 5*

1.4 Registered nursing associates' experiences tempered by professionals’ expectations in practice.

Early high expectations of the role amongst registered nursing associates were tempered by their experiences in practice. Their early optimism and expectations were not always retained. Registered nurses reported a presumption amongst colleagues that the Band 4 registered nursing associate role was ‘less safe’ than the Band 5 registered nurse and that the expectations of wards receiving registered nursing associates were ‘not usually high’. Three senior registered nurses interviewed were resolute that a registered nurse was preferable to a registered nursing associate on the ward. Their rationale was linked to workplace capacity, not individual ability:

“*I don't think we have the staffing resources to do this successfully*” Registered Nurse 16 Band 7

The registered nursing associate role was sometimes seen to diminish rather than expand nursing resources. A ward manager described the impact of registered nursing associates on other staff groups:

“*So, the* registered nurse's *workload is now increased because you must supervise that* nursing associate *[..] Why would you want a* nursing associate *when you could have a* registered nurse*? It feels dishonest*.” Registered Nurse 20 Band 8

Concerns were also voiced over the economic rationale and long-term sustainability for changes to the workforce skills mix:

“*Workforce planning - I am also talking about financial structures here, have not been really carefully thought through. How are we funding this role? We cannot rely on HEE [Health Education England] funding. [..] We are making sacrifices now which seems unfair. Band 5 [nurse] posts are removed to put in Band 4 [registered nursing associates] posts, we are not removing healthcare assistants to have [registered nursing associatess. We are reducing the balance of skill, losing registered nurses, but not getting any additional money. Can this continue in the longer term*?” Registered Nurse 20 Band 8

Some participant registered nursing associates voiced the need to “demonstrate” or “prove” their worthiness in the clinical team and some nurses observed that when registered nursing associates were introduced to their clinical areas, coworkers’ expectations changed:

“*Perhaps [..] areas should be a bit more open minded. Initially one of the [specialist] areas said no, no, no. But now they have sent an healthcare assistant into the nursing associate programme and they are doing very well” [..] They will probably all say “oh they won't be capable enough to do this”, but they have proved us wrong*" Registered Nurse 19 Band 8

2. Credibility of the registered nursing associate role in practice

The credibility of the registered nursing associate role in practice has three sub-themes, the role was perceived as (2.1) a steppingstone for progression not a distinct professional identity; and participants observed a (2.2) blurring of roles in practice; wherein (2.3) registered nursing associates adapted to performing a hybrid role.

2.1 Steppingstone for progression not a distinct professional identity.

The intended ‘bridging’ between healthcare assistant and registered nurse roles did not align with the registered nursing associate's transience in practice. For some registered nursing associates, receiving a salary during training encouraged them to progress towards nursing registration without a significant debt. The registered nursing associate role was commonly viewed as a ‘steppingstone’ journey, rather than a destination. A senior nurse observed:

*"Oh, they are all stepping up to be a registered nurse. [..] That is the sort of sentiment among all of them. So I guess, I don't know whether we will have NAs anymore"* Registered Nurse 21 Band 7

In fact, staying as a registered nursing associate for long seemed undesirable or unrealistic to registered nurses due to the limited prospects for professional development. Such a situation posed a challenge for registered nurse leaders in terms of motivating the registered nursing associate workforce should they chose not to top-up to nurse education:

“*To have a continuous development plan [..] is a challenge because there is no clear pathway out there to help you develop those skills. [..] I can't see that there is anywhere for them to go, anywhere to aspire to [..] You might not want to be a nurse, that's fine - but then you are static, stuck*” Registered Nurse 19 Band 8

The sentiment of the registered nursing associate role as ‘a means to an end’ was widely recognised. The desirability of this route to nurse status was affected by the introduction of the alternative Nursing Degree Apprenticeship scheme in 2016 in England, which led to such status earlier:

*“They had seen it [nursing associate] as a direct step towards nursing. Later […] they felt a bit duped [..] and those of them who would have had the qualifications for the [registered nurse degree] apprenticeship wanted to do that.*” Registered Nurse 11 Band 7

The unanticipated preceptorship requirements for registered nursing associates to remain in post for at least six months to a year prior to application for top-up nurse education was also unwelcomed: *“I now have to do a year's worth of preceptorship before I can move up. And I think if it wasn't for that, maybe I wouldn't be a nursing associate anymore and I'd be a registered nurse.* Registered Nursing Associate 5 Band 4 (5 months registered).

2.2 Blurring of roles in practice.

In practice, professional roles along the care continuum appeared considerably blurred. Registered nursing associates' duties overlapped with those practiced by registered nurses, with healthcare assistants in turn completing tasks typically performed by registered nursing associates. Although this flexibility made it possible to meet clinical demands, the blurring of duties was sometimes questioned and not always welcomed.

A difference in the way that registered nurses and registered nursing associates were observed to practice was in the provision of “hands-on” care. Registered nursing associates were perceived to work more collaboratively, with healthcare assistants, in performing personal care - particularly with patient washing, toileting and assisting during mealtimes. However, the notion that registered nursing associates simply bridged the gap between healthcare assistants and registered nurses was considered overly simplistic, with responsibilities and tasks flowing across the spectrum of healthcare assistants, registered nursing associates, and registered nurse roles:

“*The policy, it must take a very narrow view of the healthcare assistsnt and nursing associate role to assume that there is always a gap. It does not see it as a sort of mosaic of experience, it sees it as something else, a sort of linear continuum. That is an impossibility, anyway, policy does not understand the complexities of either role”* Healthcare Assistant 7 Band 3.

Healthcare assistants questioned the Nursing & Midwifery Council's allocation of care planning (Table 2 above) as the sole responsibility of nurses:

*“Fantasy! [*registered nursing associates*] are planning, assessing, reviewing [..] instructing, in fact, and making revisions, as they go about like [..] them [registered nurses]. To say otherwise is utter [..] rubbish and [..] unfair. If they waited for a nurse to go and do it, well, it would just [..] stand still and the patients would never [..] go home*.”. Healthcare Assistant 8 Band 2

Interestingly, healthcare assistants also reported ‘acting up’, that is, carrying out tasks commonly performed by registered nursing associates: “*I now work like a nursing associate. I can do the ECGs (electro-cardiograms), I can do the blood scan, I can do a cannula. I can do many things other healthcare assistants cannot do*” Healthcare Assistant 6 Band 3.

Systemic service pressures such as the acuity of patients, the complexity of care, shortfalls in staffing, or the lack of a formal job description, shaped the ebb and flow of care role parameters, circumstances often not willingly accepted:

*“[T]he key issue is the acuity of patients that nursing associates can or can't care for. I think they are pushed to do more than they are comfortable with because of shortages of staff [..] [T]here is a real blurring of the lines, it is "so you want to register; so you will do as you are told and get the work done*" [..] *Within our professional groups [..] boundaries have diminished or disappeared sometimes*“ Healthcare Assistant 7 Band 3

*“The last thing on anyone's minds over the last two years is where the nursing associate fits in. It is just that we are happy to have extra hands. We are just firefighting."* Registered Nurse 18 Band 8

Sometimes tensions arose from expectations on registered nursing associates to work beyond their scope of practice and they acted to police the boundaries of their role

“*It's about [..] being brave and standing up for yourself to actually [say] no, that is not within my scope. [..] [I]t has caused tensions and [..] I've upset some people [..] but [..] I'm not touching [..] stuff that I should not be touching*.” Registered Nursing Associate 4 Band 4 (9 months registered).

A mechanism debated and actioned by registered nursing associates to diffuse such tension was consistent with the concept of ‘quiet quitting’ (Boy and Surmeli 2023)—a reduction in labour to fulfil required responsibilities without spending any additional energy, time, or passion required for optimal care:

*“[W]hen I went to help as nurse in charge a few shifts ago, I made sure my stuff was done first. So historically, what I used to do is try to make sure everyone was happy, and everyone had what they needed, including my own patients. So I've been doing this thing of quiet quitting where I'm not going above and beyond, which is amazing, because I'm not getting as stressed as I used to, but horrible because I'm not doing everything I could for my patients and colleagues”.* Registered Nursing Associate 4 Band 4 (9 months registered)

Concerns about working beyond role boundaries were voiced by registered nurses uncomfortable with the workload and scope of responsibilities undertaken by registered nursing associates.

*“You don't want the [registered]nursing associates to feel short changed. I can't really expect an [registered nursing associate] to take on all these patients, can I? [..] Let her take the whole bay? [..] Since Covid it is like “she can manage the whole bay" [..] We can't just throw them in the deep end and expect them to be fine. We are talking such an enormous workload"* Registered Nurse 21 Band 7

Such frequent role blurring also fostered uncertainty across all professional groups in terms of how to differentiate registered nursing associate and registered nurse roles, (“*They [doctors] didn't have a clear concept of what the [registered nursing associate] role was, and reverted to what a nurse does”)* Registered Nurse 11 Band 7 (Participant 11)

3. Perceived organisational “blindness” to the ambiguities of the registered nursing associate role

This theme refers to the disinclination of the study organisations to openly acknowledge the low role specificity of the registered nursing associate post and the impact of this ambiguity in the workplace. It includes two sub-themes; (1) inadequate acknowledgement of registered nursing associate responsibilities (2) registered nursing associates adapted to meet expectations of performing a hybrid nurse role.

3.1 Inadequate acknowledgement of registered nursing associate responsibilities.

Registered nursing associates in both Trusts reported they were included in the electronic roster as registered nurses and frequently worked unsupervised. Virtually all interviewees felt that the pay structure for registered nursing associates was ‘dishonest’ or ‘unfair’ for their work, and they should instead receive the same pay as band 5 registered nurses. Several registered nurses called for more ‘honesty’—or a ‘spotlight’—on what was happening with the role in practice (*“It should be made clear, [..] we are training you to be a nurse through this route”* Band 8 Registered Nurse. Participant 13), while some registered nursing associates spoke of feeling slighted or exploited (*“I felt sometimes, abused, like I was used just as cheap nurse”* Registered Nursing Associate 1 Band 4 (19 months qualified).

Lack of recognition of the volume of work undertaken by NAs was often voiced. Some healthcare assistants and registered nurses argued that the extent of registered nursing associates responsibilities were ‘unacknowledged’, with some senior registered nurses comparing registered nursing associates to the now-defunct State Enrolled Nurse positions:

“*On the whole people see it as unfortunately going back to the days of when we had SENs. [State Enrolled Nurses] Over time resentment grew, especially from the enrolled nurses who saw they were doing pretty much what the registered nurses were doing but were being paid less for it. [..] I can sense that is how it will go in the future because it is like, just cheap labour, basically [..] and what is fair here?”* Registered Nurse 13 Band 8.

3.2 Registered nursing associates adapted to meet expectations of performing a hybrid nurse role.

Registered nursing associates appeared to adapt in practice to meet the expectations of working beyond their intended scope of practice. They cited instances where they validated their care decisions and actions in patients’ notes as those instructed by a registered nurse. Registered nursing associates, who openly suggested that they worked in a registered nurse capacity, felt conflicted and questioned the validity of this practice but continued in performing the required care.

*“I [..] feel, currently, admissions and discharges should not be done by us nursing associates. Like we can coordinate them and discuss it with the nurse in charge. But that final discharge [..] should be done by a nurse, surely?”* Registered Nursing Associate 4 Band 4 (9 months registered)

4. Task orientation—a trend towards ‘taskification’ within care

The term ‘task’ was consistently used across professional staff groups to describe routine care practices and to explain who undertook care activities. Senior registered nurses expressed concern that extensive focus on completing tasks in managing care, a process termed ‘taskification’ (Blanco-Mavillard et al., 2022), resulted in ill-defined responsibilities and potentially threatened patient safety if critical thinking and decision-making were not prioritised. A ward manager commented:

“*I wanted [the nursing associate] to focus on the patient care, really understanding what is going on with deteriorating patients [..] There is too much task comparison between wards where they [nursing associates] come from and what we are doing here. This is not helpful. My ward is really kind of different in that we get quite a lot of aggressive violent patients. We have to look beyond allocation of tasks. Think “when should I escalate”?* [raise concerns] Registered Nurse 19 Band 8

The emphasis on task completion was perceived by some to take priority over building supportive professional relationships in practice:

“*For me it feels unpersonal (sic) […] like its finish that one, that task, [..] Then what's the next? What's happening? whose waiting? [..]. I think, managers should ask, [..] has anything [..] disturbing happened?, anything upsetting happened? [..] Those [..] are the kinds of questions that you want to be (sic). But no. It's ‘whose bloods are done?’, ‘who is washed’?”* Registered Nursing Associate1 Band 4 (19 months qualified).

Patient-centred communication also sometimes seemed to be absent in a task-focused care environment, which led to moral distress:

``*The holistic caring, the talking, the listening, well that is not highlighted, and critical thinking is not highlighted [..] "It seems to be all about [..] getting on with tasks. The onus on a good bedside manner, really communicating with patients, fostering this dialogue is missing—in [all] our Bands [..] There is care and there is caring care. I think what has happened here is due to low resources, yes, shortages of people, of everything, and this is very sad.” Registered Nurse 11 Band 8.*

## 4 Discussion

This study revealed a mismatch between the identity of the registered nursing associate role as envisaged by the education and regulatory bodies in the UK, and its implementation and reality in practice. Although intended as a bridging role between healthcare assistants and registered nurses, the registered nursing associate seemed to predominantly function in a registered nurse capacity. Yet, registered nursing associates' desire to use the role as a steppingstone to registered nurse status was not readily attained. As early adopters, the registered nursing associates did not experience role consolidation or the availability of specialisation in their role, factors which may reduce its credibility as a career destination. Concerns voiced by registered nurses that the registered nursing associate role could replicate the limited career prospects associated with the defunct NHS state-enrolled nurse position are echoed in King et al.’s (2020) study which identified apprehension that the registered nursing associate post could be similarly restrictive.

The two NHS Trusts seemed to adopt a flexible approach to the implementation of the registered nursing associate role. Different clinical areas had different requirements and views on what registered nursing associates could do, fueled by the individual registered nurses' perceived competencies and willingness to accept tasks. The often-voiced ‘Emperor's New Clothes’ metaphor suggested a ‘blind eye’ regarding the organisational reluctance to critique or review the role in view of the flexibility it afforded. Yet the ambiguity perceived from the blurring of role responsibilities generated uncertainty and anxiety in practice, a finding mirrored in King et al.’s (2020), Coghill (2018) and Lucas et al.’s (2021) evaluations of apprentice nursing associates' and registered nursing associates' experiences.

Fotaki and Hyde (2015) identify that ‘*blind spots’* develop in organisations as a defence mechanism for coping with problems arising from attempts to implement unrealistic strategic and policy objectives. In our study, nurses may have found themselves defending or turning a blind eye to the discretionary usage of registered nursing associates in practice simply to fill gaps in services and keep practice operating safely. Yet this very usage may have increased feelings of unsafety in the participants. Moreover, it is possible that registered nursing associates felt obliged to provide care beyond their scope of practice.

Accounts of registered nursing associates' apprenticeship experiences indicate learning and development opportunities required to achieve their registration appeared to be negotiated and hard won. Nevalainen et al. (2018) identify lack of management support as an impediment to formal and informal work-based learning in health organisations. However, in this study, the challenges encountered point to a need for both higher education institutions and NHS Trusts to create a practice environment where ‘learning’ and ‘working’ are not perceived as competing priorities, but as integrated and part of a foundation to support and facilitate continuing professional development.

Registered nursing associates acculturated to fit in and meet practice demands and some employed ‘quiet quitting’ to manage distress by covertly setting boundaries. In ‘quiet quitting’ employees act exactly within their job descriptions, but without passion and work commitment. Inadequate management, emotional exhaustion, disengagement and depersonalisation underlie this withdrawal. This study suggests it may be a strategy to delineate roles. Other authors propose quiet quitting can trigger a toxic organisational culture and must be seen by leaders as a sign of moral distress (Boy and Surmeli, 2023). Jameton (1984) identified that ‘moral distress’, arises ‘*when one knows the right thing to do, but institutional constraints make it nearly impossible to pursue the right course of action’.* Those who experience moral distress can withhold care, which can impact on duration of patient stay, increased complications and mortality rates (Lamiani et al., 2017).

Boy and Surmeli (2023) propose that improved alignment of staff needs, motivations and expectations is required to support professional development and staff retention. Although it is well established that managers should provide close supervision and monitoring to support clarity, embeddedness and accountability for new roles, Lucas et al. (2021); Busca et al. (2021); Kim and Shin (2020); Halse et al. (2018), this is only feasible if there is sufficient organisational readiness and capacity to enable it. A clinical environment of increasing patient acuity, burgeoning workload and limited resources (Dunn et al., 2023) is unlikely to facilitate role implementation and sustain the required levels of support for registered nursing associates. The preoccupation with ‘tasks’ in the two NHS Trust may signal a ‘firefighting’ response (McKeown et al., 2019) where efforts to contain service pressures do not address the underlying shortage of resources fueling these challenges.

A tension seemed to exist wherein the difference between ‘nursing’ and ‘nurses’ per se was not recognised. The rationale presented by most study participants that registered nursing associates deserved more recognition and pay as they ‘did’ the same ‘tasks’ as nurses may fail to recognise that the expectations from multiple stakeholders (i.e., the UK Nursing & Midwifery Council) , their own profession, other health and non-health professionals, and the public) for nurses regarding their decision-making and understanding of holistic and pre-emptive care would be different than the expectations of registered nursing associates (Steven et al., 2023). Perhaps concerningly, these differences were not readily apparent in this study. At the heart of all this is a discussion about what extra contributions nursing workforce professionals bring to their roles over and above the performance of set tasks, which others may perform just as well or better. Further exploration and definition of these aspects of caring are required, including any externalities and unintended consequences.

## 5 Limitations

This study has several limitations. It was undertaken in two London (UK) acute Trusts with high rates of nursing vacancies, at a time when staff were still recovering from the COVID-19 pandemic. The responses might have been given with social desirability in mind. In addition, we do not know whether participants claiming that responsibilities of nursing associates were unclear had read any documents available to support role realisation. A balance in representation across and within professional groups was not achieved. The higher concentration of predominately senior nurses reflects who came forward for interview. Senior managers, education leaders, or other organisational perspectives were not examined, nor were organisational guidelines or registered nursing associate implementation strategies.

## 6 Conclusion

The views and experiences of registered nursing associates, healthcare assistants and registered nurses in this study raise some doubt around the effective implementation of the registered nursing associate role. Perceived organisational expectations about the scope of practice of registered nursing associates differed from those held by registered nursing associates, who largely aspired to progress to become registered nurses. Registered nursing associates were commonly perceived by healthcare assistants and registered nurses to be working as substitute nurses. This fluidity around the application of the registered nursing associate role appears to contribute to a sense of invisibility and lack of professional identity.

Efforts must now be made to prioritise registered nursing associate’s professional development rather than simply engaging their capacity to plug gaps in service delivery. However, unless consensus exists in terms of the duties and responsibilities of registered nursing associates, efforts to develop the role to support post-holders’ progression will have limited relevance. Furthermore, as the role gains momentum and extension in practice, further research and evaluation is needed to ensure that pre and post registration education and practice support meets and balances personal professional and service needs. Finally, it is essential that the psychological and moral distress associated with the gap between experiences and expectations is listened to and addressed.

## Funding

This project was funded by Imperial Health Charity / NIHR Imperial BRC within a pre-doctoral award for the first author. The funders were not involved in data collection, analysis or writing of this paper.

## Data not available/The data that has been used is confidential

Due to the confidential nature and potentially sensitive nature of the questions asked in the study interviews participants were assured that the data collected would remain confidential and would not be shared.

## CRediT authorship contribution statement

**Carolyn Spring:** Conceptualization, Validation, Methodology, Data curation, Formal analysis, Investigation, Software, Visualization, Writing – original draft, Project administration, Funding acquisition. **Enrique Castro-Sánchez:** Conceptualization, Supervision, Formal analysis, Writing – review & editing, Resources, Validation. **Mary Wells:** Conceptualization, Supervision, Formal analysis, Writing – review & editing, Resources, Validation.

## Declaration of competing interest

The authors declare that they have no known competing financial interests or personal relationships that could have appeared to influence the work reported in this paper.

## Appendix Supplementary materials

Image, application 1
