# Supplementary material for: ‘The Emperor's new clothes?’ Healthcare professionals’ perceptions of the nursing associate role in two UK National Health Service hospitals: A qualitative interview study
Source: Int J Nurs Stud Adv. 2024 Jun 6;7:100211. doi: 10.1016/j.ijnsa.2024.100211 (PMC11334778; doi:10.1016/j.ijnsa.2024.100211)
Supplement: Supplementary file 1 [file mmc1.docx]

*Data not available / The data that has been used is confidential*

Due to the confidential nature and potentially sensitive nature of the questions asked in the study interviews participants were assured that the data collected would remain confidential and would not be shared.
